# Association Between Healthy Lifestyle and Cardiometabolic Risk in Higher Education Students in a Region of Peru: A Cross-Sectional Survey

**DOI:** 10.3390/nu17243944

**Published:** 2025-12-17

**Authors:** Saulo A. Salinas Arias, Wildoro Ramírez Ramírez, Eliseo Alava Peña, Ledmy Vásquez Ruiz, Norma L. Alejandría Lozano, Jhoel A. Llique Tanta, Jania E. Jaimes Soncco, Jessica Pérez Rivera, Jacksaint Saintila, Wilter C. Morales-García

**Affiliations:** 1 Professional School of Psychology, Universidad Peruana Unión, Tarapoto 22201, Peru; jani@upeu.edu.pe; 2 Department of Humanities and Social Sciences, Universidad Nacional de San Martín, Tarapoto 22201, Peru; wramirez@unsm.edu.pe (W.R.R.); lvasquez@unsm.edu.pe (L.V.R.); 3 Unidad de Gestión Educativa Local San Martín, Tarapoto 22201, Peru; eliseoalavapena@gmail.com; 4 Unidad de Bienestar y Empleabilidad, Escuela de Educación Superior Pedagógica Pública, Tarapoto 22201, Peru; josemaria_200@hotmail.com; 5 Facultad de Educación Física y Deportes, Universidad Nacional de Educación Enrique Guzmán y Valle, Lurigancho-Chosica, Lima 15, Peru; jllique@une.edu.pe; 6 Professional School of Civil Engineering, Universidad Peruana Unión, Tarapoto 22201, Peru; jessica.perez@upeu.edu.pe; 7 Research Group for Nutrition and Lifestyle, School of Medicine, Universidad Señor de Sipán, Chiclayo, Lambayeque 14001, Peru; 8 Dirección General de Investigación, Universidad Peruana Unión, Lima 15, Peru; wiltermorales@upeu.edu.pe

**Keywords:** cardiometabolic risk, lifestyle, obesity, waist-to-height ratio, university students, Peru

## Abstract

**Background:** Cardiometabolic diseases are among the leading causes of mortality worldwide and are strongly influenced by lifestyle factors. Objective: The aim of this study was to determine the association between a healthy lifestyle and cardiometabolic risk in higher education students in the San Martín region, Peru. **Methods:** A cross-sectional study with non-probabilistic convenience sampling was conducted among 1054 students from higher education institutions in the San Martín region, Peru. The Healthy Diet and Lifestyle Scale (DEVS) was applied, and anthropometric indicators (BMI and waist circumference) were assessed by trained nutritionists. Descriptive statistics, association tests (chi-square and Wilcoxon), and Poisson regression models with robust variance were used. **Results:** Obesity was significantly associated with higher vitamin B12 intake (PR = 2.39; 95% CI: 1.16–4.91) and with higher water consumption (>8 glasses/day) (PR = 2.61; 95% CI: 1.20–5.66), although these findings may reflect reverse causality given the cross-sectional nature of the data. Greater whole grain consumption was associated with a lower risk of elevated waist circumference (PR = 0.60; 95% CI: 0.40–0.91). Similarly, engaging in ≥30 min of daily physical activity was significantly associated with reduced central adiposity (PR = 0.69; 95% CI: 0.56–0.86). **Conclusions:** The findings suggest that whole grain consumption and regular physical activity act as protective factors, whereas certain dietary patterns—despite being considered healthy—may be associated with higher cardiometabolic risk depending on the dietary context.

## 1. Introduction

Cardiometabolic diseases (hypertension, diabetes, dyslipidemias, and obesity) [1] encompass a range of conditions that affect the metabolic and cardiovascular systems [2], including coronary heart disease and stroke [3]. These diseases are the leading cause of death in the Americas [4]. Each year, more people die from these conditions than from any other cause, and their prevalence continues to rise in low- and middle-income countries [4]. A study conducted in Peru between 2015 and 2023 analyzed data from the Demographic and Family Health Survey (ENDES), which is carried out annually on a nationally representative sample [5]. They concluded that the prevalence of high blood pressure increased between 2015 and 2017 and between 2020 and 2023, with the greatest increases observed in the overweight and obese groups; conversely, the prevalence of self-reported diabetes increased in all BMI categories [6]. However, since these diseases are preventable, counteracting these trends requires not only addressing lifestyle factors but also developing immediate intervention strategies [7].

A healthy lifestyle is defined as a set of daily behaviors and choices that contribute to overall well-being and optimal health [8]. These behaviors include a variety of practices and habits such as diet, regular physical activity, adequate water intake, sleep quality, sunlight exposure, and others [9]. Although these choices are voluntary, they determine health status in the medium and long term. Thus, educational institutions could serve as strategic allies in promoting students’ healthy lifestyles [10]. However, adherence to a healthy lifestyle can be challenging during youth, largely due to environmental conditions and characteristics [11]. One such factor is parental absence, as a significant portion of students leave their homes to pursue higher education, gaining autonomy that often leads them to choose, out of necessity, less healthy foods. This tends to result in a preference for foods high in fats and sugars [12], along with a low intake of fruits and vegetables [13]. Peru is no exception: according to the National Institute of Statistics and Informatics (INEI), the daily consumption of fruits and vegetables among individuals over 15 years of age is only two servings [14], and this insufficient intake may contribute to the development of cardiovascular diseases, cancer, diabetes, and obesity [14].

On the other hand, there is an increase in overweight, obesity, and alcohol consumption among students in higher education institutions. This has been evidenced in a study conducted with Jordanian university students, where one-third of the students were found to be overweight or obese and displayed unhealthy lifestyle behaviors [15]. Similar findings were reported among Iranian university students, who showed a prevalence of overweight and abdominal obesity, particularly among males [16]. In Spanish students, an association was observed between obesity and certain unhealthy lifestyle patterns [17], while a study at a Russian university revealed that 30% of the students were overweight and more than 54% reported alcohol abuse [18].

Meanwhile, cardiometabolic risks may be influenced by both genetic factors and lifestyle behaviors [19], one of which is physical inactivity—the fourth most common risk factor for death worldwide, responsible for approximately 3.2 million deaths each year [20]. This was highlighted in a study of Italian students, which found a direct association between very low physical activity frequency and higher body mass index values [20]. In Colombia, excess weight was associated with poor eating habits, physical inactivity, and alcohol consumption [11]. It was also observed that the highest scores were found among those with a BMI greater than 25, lower levels of physical activity, inadequate fruit and vegetable intake, and higher body fat mass [21].

It is worth noting that adopting a healthy lifestyle can significantly reduce the risk of developing cardiovascular and metabolic diseases among higher education students. Therefore, this study is both important and relevant, as this stage of life represents a critical period for fostering new cultures, dietary practices, and lifestyle habits [22]. However, evidence on the associations between lifestyle and cardiometabolic risk among higher education students in developing countries, such as Peru association remains. Thus, the present study aims to determine the association between a healthy lifestyle and cardiometabolic risk among higher education students in the San Martín region of Peru.

## 2. Materials and Methods

### 2.1. Study Design and Participants

This quantitative, associative, cross-sectional study was conducted between May and June 2024. It was carried out in higher education institutions (HEIs) in the San Martín region of Peru, five of which were public and two private. Students were recruited through a non-probabilistic convenience sampling approach. Coordinators and faculty from each HEI facilitated access to classrooms and common areas, where trained nutritionists approached students in person, briefly explained the study objectives, eligibility criteria, and voluntary nature of participation. Data collection took place in designated spaces within each institution (e.g., classrooms, auditoriums, and student service areas) to ensure privacy and adequate logistical conditions. Students who agreed to participate completed the survey and anthropometric assessment during a single session lasting approximately 20 min. Participants included students enrolled in the first academic semester of 2024, from the first to the fifth year of study and representing diverse technical and university programs.

The survey consisted of 25 items, eight of which addressed sociodemographic aspects, three assessed cardiometabolic risk (BMI and waist circumference), and 14 evaluated healthy lifestyle. To minimize bias and error during data collection, interviewers were trained to record the participants’ responses. At the same time, participants were reminded of the importance of providing accurate and relevant information. Detailed and standardized instructions were also provided for anthropometric measurements.

A total of 1650 students aged 16 to 23 years were initially considered for inclusion. However, the following participants were excluded: 110 students under 18 years of age who did not provide assent, 85 participants aged 18 or older who did not provide informed consent, 170 students who declined to participate, 140 students who submitted incomplete questionnaires, and 91 students with missing anthropometric measurements. After applying these exclusion criteria, a total of 1054 participants were included in the final analytical sample.

The sample size was calculated using Free Statistics Calculators, version 4.0 (Soper) [23]. Although the primary analyses were performed using Poisson regression models with robust variance, the required sample size was estimated using Cohen’s *f*^2^ for multiple regression (*f*^2^ = 0.02), assuming two main predictors, a statistical power of 0.80, and a significance level of 0.05. This approach provides a conservative estimate and is commonly applied when planning multivariable models assessing associations between lifestyle factors and health outcomes. The calculation indicated that 478 participants would be sufficient [24]. However, to increase the robustness and validity of the results, a larger sample size was considered for this study, reaching a total of 1054 participants.

### 2.2. Ethical Considerations

Written informed consent was obtained, ensuring that students had voluntarily participated and had a clear understanding of their involvement in the study. Ethical approval for the research protocol was granted by the Research Ethics Committee of Universidad Peruana Unión under the approval number 2024-CEUPeU-021. The study was conducted in strict accordance with international ethical standards, following the principles set forth in the Declaration of Helsinki and its subsequent amendments.

#### Variables

Lifestyle. To assess a healthy lifestyle, we used the Healthy Diet and Lifestyle Scale (DEVS) [23], the Peruvian version of the instrument, which was initially validated by Le and colleagues [11] under the name of the Vegetarian Lifestyle Index (VLI). The validation of the DEVS, similar to the VLI, consists of 14 items [25]. Eleven of these items address aspects related to a diet based on the consumption of whole plant-based foods such as fruits, legumes, seeds, and cereals, as well as animal-derived foods, including milk, dairy products, eggs, and reliable sources of vitamin B-12, along with one question about sweets and candies. In addition, the last three questions focused on physical activity, water intake, and sunlight exposure [26]. All items used a three-point ordinal response scale scored as integers (0, 1, or 2). For the reverse-coded components (vegetable oils, dairy products, eggs, sweets, and meat intake), the raw responses were recoded so that higher consumption received a lower score (e.g., 2 → 0; 1 → 1; 0 → 2). No weighting system was applied; instead, the final lifestyle score was obtained by summing the 14 integer-coded items, producing a total score ranging from 0 to 28. In our sample, the DEVS showed good internal consistency, with a Cronbach’s alpha of 0.81, indicating adequate reliability for research use. For analytical purposes, the lifestyle score was dichotomized using the median value (<27 vs. ≥27), which allowed for adequate group differentiation in the regression models.

BMI. Weight and height measurements were obtained using the SECA (Hammer Steindamm 3-25 22089, Hamburg, Germany) scale and a stadiometer with high precision. During the assessment, participants wore minimal clothing and no shoes. BMI was subsequently calculated using the WHO formulas, and the following classification was applied: <18.5 underweight, 18.5–24.9 normal, 25–29.9 overweight, and ≥30 obesity [27].

Cardiometabolic risk. Cardiometabolic risk (CMR) was determined by measuring waist circumference (WC) using a self-retracting steel tape measure (Cescorf Equipamentos Para Esporte Ltda-Epp, Porto Alegre - RS, 91900-050, Brazil). CMR was defined as a waist circumference ≥ 94 cm in men and ≥80 cm in women. These parameters were established according to the Peruvian Technical Guide for the Nutritional Anthropometric Evaluation of Adults [27].

Sociodemographic Information. Sociodemographic data were collected through a set of eight questions covering various categories, including age, sex, year of study, and educational institution. Participants were also asked, “Who do you live with?” and how many siblings they had at the time of the study. In addition, information was collected on the parents’ educational level and socioeconomic status.

### 2.3. Statistical Analysis

Sociodemographic, anthropometric, and lifestyle characteristics were described using means and standard deviations for numerical variables, and frequencies for categorical variables. Lifestyle factors, both individual items and the overall score, were compared according to obesity and elevated waist circumference. The chi-square test of independence and the Wilcoxon rank-sum test were used to compare categorical and numerical variables, respectively. Poisson regression with robust variance was then applied to calculate prevalence ratios (PRs) with 95% confidence intervals (95% CI) for the association between lifestyle and two dependent variables: obesity and elevated waist circumference. Regressions were performed both unadjusted and adjusted for sex, educational level, socioeconomic status, physical activity, and age. Variables with a *p*-value less than 0.05 in the adjusted regression were considered statistically significant. All participants with incomplete questionnaire responses or missing anthropometric measurements were excluded before analysis, as detailed in the participant flow.

## 3. Results

As shown in Table 1, the study sample consisted of 1054 university students, with a slight majority being women (55.7%). The mean age was 18.7 years with a standard deviation of 1.0. Regarding the year of study, most participants were in their first year (64.4%). The majority of students attended public institutions (64.3%). The average number of siblings was 2.7 with a standard deviation of 1.7. In terms of living arrangements, the largest proportion lived with both parents (43.5%). Concerning parents’ educational level, only 11.1% had a university degree. Finally, the predominant socioeconomic level was the lowest category (1300 PEN), representing 65.7% of the sample.

Table 2 presents the anthropometric characteristics of the participants. The mean weight was 60.9 kg (±11.7), while the mean height was 1.6 m (±0.1). The average BMI was 23.5 (±3.8). Approximately 93.9% of the participants were non-obese, whereas 6.1% were classified as obese. Regarding waist circumference, 63.5% of the students had a normal waist circumference, while 36.5% had an altered waist circumference, which could indicate a higher risk of metabolic complications. When stratified by sex, 27.2% of men exhibited an altered waist circumference compared to 43.9% of women, indicating a higher prevalence of central adiposity among female students.

Table 3 presents the lifestyle characteristics of the participants. Meat and processed food consumption was the most prevalent, reported by 47.5% of students. A low frequency of consumption was also observed for foods considered healthy, such as whole grains (7.0%), legumes (19.6%), nuts (6.2%), and vegetable oils (8.2%). These dietary patterns reflect a tendency toward frequent consumption of processed foods and an insufficient intake of foods recommended for a balanced diet.

Table 4 shows lifestyle according to BMI status. Significant differences were observed between obese individuals and water intake (*p* = 0.006), as well as reliable sources of vitamin B12 (*p* = 0.005). However, most lifestyle-related variables did not show statistically significant differences.

Table 5 presents the lifestyle according to elevated waist circumference. Significant differences were observed (*p* = 0.009) in the number of whole grain portions consumed per day, with a higher proportion of participants classified with elevated waist circumference (≥94 cm in men and ≥80 cm in women) among those who consumed fewer than three portions of whole grains per day. Regarding physical activity, the data revealed significant differences (*p* < 0.001), with a higher percentage of participants with normal waist circumference among those who reported not engaging in physical activity or engaging in less than 30 min per day.

Table 6 shows the association between lifestyle factors and obesity, where a significant association (*p* = 0.032) was observed for legume consumption, indicating a lower likelihood of obesity. Likewise, higher intake of vitamin B12 sources (meat, fish, dairy products, eggs, fortified foods, and supplements) showed a significant association (*p* = 0.018). In addition, consuming more than eight glasses of water per day was significantly associated with obesity (*p* = 0.015). Meanwhile, higher consumption of sweets was related to an increased probability of obesity, although this association did not reach statistical significance (*p* = 0.054).

The association between lifestyle components and elevated waist circumference is presented in Table 7. A higher intake of whole grains was significantly associated with a lower prevalence of elevated waist circumference, with a PR of 0.84 (95% CI: 0.71–0.99; *p* = 0.040). In contrast, not engaging in physical activity or engaging in less than 30 min per day was significantly associated with a higher prevalence of elevated waist circumference, with a PR of 1.22 (95% CI: 0.83–1.80; *p* < 0.001). None of the other dietary or lifestyle components showed statistically significant associations (*p* > 0.05), suggesting that these behaviors may not be strongly related to the outcome in this sample.

## 4. Discussion

In this study, it is confirmed that despite a low prevalence of obesity according to body mass index (BMI), a relevant proportion of students present an elevated waist circumference, suggesting that cardiometabolic risk may be underestimated when relying solely on BMI for screening in higher education [28,29,30]. In our analyses, central adiposity was assessed using waist circumference rather than the waist-to-height ratio (WHtR); therefore, all reported associations refer to this indicator. This finding aligns with evidence that central adiposity markers (waist circumference or WHtR) discriminate cardiometabolic risk better than BMI alone and with proposals that emphasize keeping waist measures within recommended cut-off points as a simple public health message [28,30,31]. Studies in university and general populations, including Peruvian samples, also indicate that elevated central adiposity is associated with higher cardiometabolic risk even among individuals with BMI in the non-obese range [32,33,34]. Given that visceral adiposity contributes to inflammation, insulin resistance, and atherogenic dyslipidemia that may not be captured by BMI, the “normal weight with central adiposity” phenotype represents an underdetected risk profile when only weight and height are used [26,27]. Taken together, these results support the combined use of BMI and simple central adiposity measures, such as waist circumference, to improve the identification of young adults at higher cardiometabolic risk [27,28].

Behavioral factors typical of university life (intermittent sedentary behavior, irregular eating patterns, insufficient sleep) and urban environments with high availability of ultra-processed foods may promote central fat accumulation regardless of total body weight, while contextual determinants such as socioeconomic status, sex, and residential altitude can modulate adiposity distribution and, consequently, the performance of each anthropometric index [31,32,35]. Although we did not measure these mechanisms directly, previous evidence supports their role in shaping abdominal fat distribution and cardiometabolic risk in young populations [31,32,35].

The findings also reveal a nutritional paradox among higher education students: despite a low prevalence of obesity according to BMI, the observed dietary pattern tends toward frequent consumption of meat and processed foods, with limited presence of whole grains, legumes, nuts, healthy oils, and vegetables. This suggests a dietary quality below international recommendations and a latent cardiometabolic risk that may not be captured when relying solely on body weight [36,37]. This pattern is consistent with literature from diverse university settings reporting high availability and preference for fast preparations and ultra-processed products, accompanied by low intake of fruits, vegetables, and whole grains [38,39,40]. Greater exposure to ultra-processed foods—often energy-dense and high in sodium, unfavorable fats, and free sugars—tends to displace protective foods, whereas a low intake of minimally processed plant foods reduces fiber and micronutrient density. Within university food environments that prioritize convenience and quick purchases, these conditions may help explain the persistence of unfavorable dietary patterns among students [36,41].

The convergence of these factors is in line with evidence linking higher consumption of red and processed meats to an increased risk of type 2 diabetes and other cardiometabolic outcomes, as well as with studies that highlight the benefits of dietary patterns rich in whole grains, legumes, nuts, and vegetables—core components of international guidelines—for improving diet quality and cardiometabolic profiles [36,38,42,43]. In this context, our findings suggest that risk assessment in young adults should consider both anthropometric indicators and dietary quality, since a pattern characterized by a high proportion of meats and processed foods and a low density of protective foods may be associated with less favorable cardiometabolic trajectories even in the absence of excess body weight. However, these interpretations are associative rather than causal and should be viewed as hypothesis-generating given the observational and cross-sectional design.

In higher education students, the positive association between legume consumption and obesity, as well as the greater risk observed with a diet richer in vitamin B12–containing foods, suggests that the effect of these groups depends less on the “healthy label” of the food itself and more on the dietary pattern in which they are contextualized—particularly the energy density of the meal, cooking techniques, and accompanying foods. In this framework, legumes may be functioning as markers of energy-dense preparations or as substitutes for other protective groups, while higher intake of animal-source foods, although providing micronutrients such as B12, tends to converge with patterns high in fats and calories, which are compatible with greater adiposity [44,45,46].

The direction of the association contrasts with much of the evidence linking legumes to less weight gain and improved cardiometabolic profiles, as well as with research showing that higher overall diet quality—characterized by greater legume intake and lower red and processed meat consumption—is associated with more favorable weight trajectories [44,47]. Cohort studies have also reported that greater intake of red and processed meats is associated with long-term weight gain, offering a plausible explanation for the finding regarding animal-based B12-rich foods [45]. A possible explanation is that culinary practices may attenuate or reverse the potential beneficial effects of legumes when they are prepared with solid animal fats or combined with refined accompaniments, thereby increasing the energy density of the dish; such practices have been documented in traditional recipes where legumes are cooked with lard, increasing the saturated fat content of the final preparation [46,47]. In addition, part of the benefit of legumes relies on their contribution of fiber and resistant starch; when the overall dietary pattern is low in fiber, the protective effect may be diluted, as suggested by the attenuation of associations after adjusting for dietary fiber in previous studies [42]. Similarly, a higher presence of B12-rich foods may be indicative of omnivorous patterns with high intakes of meat and full-fat dairy, which are energy-dense and have been associated with weight gain in prospective studies [45,46]. These explanations are inherently speculative and cannot be confirmed with our data because we did not measure total energy intake or detailed culinary practices; therefore, they should be interpreted as hypotheses that require confirmation in future longitudinal or experimental research.

This study also found an unexpected association between higher water intake and obesity. A likely explanation is reverse causality, whereby individuals with obesity increase their water consumption as a compensatory behavior after receiving health advice or in response to weight concerns. Self-report bias may additionally contribute if participants with obesity overestimate their water intake due to social desirability or misestimation of portion sizes. Residual confounding by other unmeasured behaviors (e.g., substitution of sugar-sweetened beverages, higher intake of energy-dense foods despite drinking more water) may also play a role. Taken together, these considerations suggest that the observed association between water intake and obesity is more plausibly a marker of behavioral change or reporting patterns among students with higher adiposity than a harmful effect of water consumption itself.

Our results also show that higher consumption of whole grains is associated with a more favorable abdominal adiposity profile, and that regular physical activity is linked to a lower likelihood of elevated waist circumference, converging into a cardiometabolic protective pattern among higher education students [48,49,50]. These findings are consistent with evidence linking diets rich in fiber and whole grains to lower central adiposity and improved metabolic control, as well as evidence relating habitual exercise to more favorable adiposity markers and reduced risk of abdominal obesity in young university and adult populations [51,52,53,54]. From a biological perspective, whole grains provide fiber and food matrices that modulate glycemic response, increase satiety, promote the production of short-chain fatty acids, and may enhance fecal energy loss and resting energy expenditure, which have been proposed as potential pathways underlying their protective effect against abdominal fat accumulation [51,52,55]. In parallel, regular physical activity improves insulin sensitivity, stimulates lipolysis in visceral adipose tissue, and contributes to sustained negative energy balance; moreover, in the university context, it reduces stress and improves sleep, behavioral factors that often exacerbate the intake of energy-dense foods and the risk of central adiposity [49,53].

Among students, the combination of irregular schedules, food environments with high availability of ultra-processed products, and academic sedentarism tends to displace healthy eating and movement patterns. Therefore, prioritizing whole grains as the main carbohydrate source and institutionalizing opportunities for curricular and extracurricular physical activity may represent plausible strategies to reduce the risk of abdominal obesity on campus [50,54]. Although the direction of the associations is consistent with the literature, meta-analyses of trials have shown heterogeneity and, in some cases, null effects of whole grains on global obesity markers, underscoring the importance of considering the quality of whole-grain foods, adherence, and outcomes centered on abdominal adiposity rather than total weight. Moreover, longitudinal and experimental designs in university populations are needed to integrate behavioral and metabolic mediators to clarify the magnitude and sustainability of the observed effects [56,57].

This study has several limitations that should be considered when interpreting the findings. First, we did not assess total energy intake, specific food groups in grams, or detailed cooking methods, which limits our ability to evaluate misclassification of dietary exposures and to test mechanistic pathways related to energy balance. Second, diet and lifestyle variables were based on self-report, which is susceptible to recall and social desirability bias and may have led to misclassification of consumption categories. Third, the set of covariates was restricted; residual confounding by unmeasured factors such as socioeconomic gradients, psychosocial stress, or sleep patterns cannot be ruled out. Fourth, we relied on waist circumference as the sole measure of central adiposity and did not include objective assessments of body composition (e.g., dual-energy X-ray absorptiometry) or additional indices of fat distribution, which may provide a more comprehensive characterization of adiposity. Finally, all measurements were collected at a single time point in a cross-sectional design, which precludes establishing temporal relationships between lifestyle factors and adiposity and limits causal inference.

## 5. Limitations and Future Research

This study has several limitations. First, its cross-sectional design precludes establishing temporal or causal relationships; the observed associations—particularly the positive relationship between higher water intake and adiposity—may reflect reverse causality or self-report bias rather than direct effects. Second, lifestyle data were captured with a brief instrument and coarse frequency categories, which may lead to exposure misclassification (e.g., heterogeneity in the quality of “whole-grain” items, preparation methods for legumes, or fat content of animal-source foods). Third, total energy intake, alcohol, smoking, sleep, and stress were not comprehensively measured, leaving room for residual confounding. Fourth, the sample was non-probabilistic and region-specific (San Martín), which limits generalizability to other student populations and settings; seasonality was not assessed. Finally, central adiposity was operationalized using waist circumference cut-offs only, and biochemical markers (lipids, glucose/insulin, inflammatory markers) and objective body composition measures were not collected, restricting mechanistic interpretation and the ability to characterize adiposity in greater detail.

Future research should prioritize longitudinal cohorts and pragmatic trials to evaluate campus-level interventions that simultaneously modify the food environment and opportunities for physical activity. Natural experiments (e.g., procurement changes, pricing or labeling policies) and randomized programs to increase whole-grain availability/substitution and to reduce ultra-processed foods could provide stronger evidence for causal links. Studies should also integrate high-resolution dietary assessment (multiple 24 h recalls, food processing level, culinary techniques), device-based physical activity/sleep measures, and biomarkers of metabolic health and inflammation. Mediation and effect-modification analyses (e.g., by sex, socioeconomic status, altitude) may help clarify pathways via fiber, energy density, and behavioral mediators (sleep, stress), while mixed-methods work—including qualitative research on cafeteria choices and cooking practices—can illuminate context-specific barriers and facilitators to healthier patterns.

## 6. Implications for Public Health

The findings of this study underscore the need to move beyond BMI-only screening in young adults and to incorporate central adiposity metrics such as waist circumference or waist-to-height ratio into student health evaluations. Such combined screening strategies may allow earlier identification of individuals who appear “normal weight” but present excess abdominal fat, thereby reducing underestimation of cardiometabolic risk at the population level.

At the population level, higher-education institutions represent a strategic setting for health promotion because they can influence both the food environment and opportunities for physical activity. Reforming campus food services to improve the availability and affordability of whole grains, legumes, nuts, vegetables, and healthy oils, while gradually limiting ultra-processed foods, may help align daily choices with international dietary standards. Complementary strategies, such as nutrition labeling at the point of purchase, healthier menu defaults, and pricing incentives for protective foods, could further guide students toward more healthful decisions.

Culinary quality also plays a role. Training for food-service staff to reduce the use of solid animal fats in legume-based dishes, together with student-oriented educational activities, can prevent the dilution of potential benefits from traditionally healthy foods. Simultaneously, policies to strengthen curricular and extracurricular physical activity—through structured programs, active commuting supports, and accessible recreational facilities—may contribute to higher levels of regular movement, which is essential for reducing central adiposity and mitigating related risks.

Finally, communication strategies should address behavioral and cultural factors. Campaigns that raise awareness about hydration, balanced dietary patterns, stress management, and adequate sleep can reduce compensatory behaviors and the misinterpretation of “healthy labels”. Tailoring interventions with an equity perspective—ensuring that protective foods remain affordable and culturally relevant—may maximize their reach and potential impact.

## 7. Conclusions

In a large sample of Peruvian higher-education students, central adiposity was common despite low obesity by BMI. Higher whole-grain intake and regular physical activity were associated with lower probability of elevated waist circumference, whereas patterns richer in B12-containing animal foods—and higher reported water intake—were linked to higher obesity risk, likely reflecting broader dietary context and possible reverse causality. Overall, risk appears to hinge less on isolated “healthy” items than on the pattern in which foods are consumed and prepared. Because of the cross-sectional design, these associations cannot be interpreted as temporal or causal relationships. Campus-level, multi-component interventions that reshape food availability and promote daily physical activity, coupled with screening that includes a central adiposity metric, may represent feasible steps to address early cardiometabolic risk in this population.

## Figures and Tables

**Table 1:** Sociodemographic characteristics of the participants.

Characteristic	n (%)/Mean (SD)
**Sex**	
Male	467 (44.3)
Female	587 (55.7)
**Age in years (M ± SD)**	18.7 (1.0)
**Year of study**	
1st	679 (64.4)
2nd	229 (21.7)
3rd	140 (13.3)
4th	3 (0.3)
5th	3 (0.3)
**Educational institution**	
Private	377 (35.7)
Public	677 (64.3)
**Number of siblings (M ± SD)**	2.7 (1.7)
**Living arrangements**	
With both parents	459 (43.5)
With father	44 (4.2)
With mother	157 (14.9)
Alone	274 (26.0)
Other relatives	120 (11.4)
**Parents’ education**	
None	29 (2.8)
Primary	259 (24.6)
Secondary	455 (43.2)
Technical	194 (18.4)
University	117 (11.1)
Parents’ socioeconomic level (PEN *)	
1300	693 (65.7)
2480	240 (22.8)
3970	68 (6.5)
7020	39 (3.7)
12,600	14 (1.3)

**Note.** M = mean; SD = standard deviation; * Values expressed in Peruvian soles (PEN).

**Table 2:** Anthropometric characteristics of the study participants.

Characteristic	n (%)/Mean (SD)
**Weight (kg)**	60.9 (11.7)
**Height (m)**	1.6 (0.1)
**BMI (M ± SD)**	23.5 (3.8)
**Final obesity status**	
Non-obese	990 (93.9)
Obese	64 (6.1)
**Waist circumference (cm)**	78.3 (9.1)
**Waist circumference**	
Normal	669 (63.5)
Altered	385 (36.5)
**Waist circumference by sex**	
**Men (n = 467)**	
Normal	340 (72.8)
Altered	127 (27.2)
**Women (n = 587)**	
Normal	329 (56.1)
Altered	258 (43.9)

**Note.** M = mean; SD = standard deviation.

**Table 3:** Dietary and Lifestyle Components of the Participants.

Characteristic	n (%)/Mean (SD)
**Whole grains (daily portions)**	
<3 portions/day	562 (53.3)
3–5 portions/day	418 (39.7)
≥6 portions/day	74 (7.0)
**Legumes and derivatives (daily portions)**	
<1 portion/day	323 (30.6)
1–2 portions/day	524 (49.7)
≥3 portions/day	207 (19.6)
**Vegetables (daily portions)**	
<4 portions/day	368 (34.9)
4–7 portions/day	445 (42.2)
≥8 portions/day	241 (22.9)
**Fruits (daily portions)**	
<3 portions/day	298 (28.3)
3–5 portions/day	443 (42.0)
≥4 portions/day	313 (29.7)
**Nuts and seeds (weekly portions)**	
<4 portions/week	806 (76.5)
4 portions/week—1 portion/day	183 (17.4)
≥1.5 portions/day	65 (6.2)
**Vegetable oils, avocado, olives (daily portions)**	
≤2 portions/day	617 (58.5)
>2–4 portions/day	351 (33.3)
>4 portions/day	86 (8.2)
**Dairy products (daily portions)**	
None	202 (19.2)
≤2 portions/day	605 (57.4)
>2 portions/day	247 (23.4)
**Eggs (daily portions)**	
None	82 (7.8)
≤1 portion/day	554 (52.6)
>1 portion/day	418 (39.7)
**Sweets (weekly portions)**	
<2 portions/week	505 (47.9)
2–5 portions/week	403 (38.2)
>5 portions/week	146 (13.9)
**Reliable sources of vitamin B12 (daily portions)**	
<1 portion/day	224 (21.3)
1 portion/day	509 (48.3)
≥2 portions/day	321 (30.5)
**Meat consumption (weekly frequency)**	
None	60 (5.7)
<1 time/month to 1 time/week	493 (46.8)
≥2 times/week	501 (47.5)
**Physical activity (daily minutes)**	
None	250 (23.7)
<30 min moderate/< 15 min vigorous	434 (41.2)
≥30 min moderate/≥ 15 min vigorous	370 (35.1)
**Water consumption (250 mL glasses/day)**	
<4	213 (20.2)
4–7	516 (49.0)
≥8	325 (30.8)
**Sunlight exposure (daily minutes, 11 a.m.–1 p.m.)**	
<5 min/day	347 (32.9)
5–9 min/day	420 (39.8)
≥10 min/day	287 (27.2)
**Healthy lifestyle score (M ± SD)**	26.8 (3.9)
**Categorical lifestyle score**	
<27	514 (48.8)
≥27	540 (51.2)

Note. M = mean; SD = standard deviation.

**Table 4:** Distribution of Lifestyle Components According to BMI Categories.

Characteristic	Non-Obese	Obese	*p*-Value ^2^
**Whole grains (daily portions)**	n (%)	n (%)	0.824
<3 portions/day	528 (94.0)	34 (6.0)	
3–5 portions/day	391 (93.5)	27 (6.5)	
≥6 portions/day	71 (95.9)	3 (4.1)	
**Legumes (daily portions)**			0.100
<1 portion/day	311 (96.3)	12 (3.7)	
1–2 portions/day	486 (92.7)	38 (7.3)	
≥3 portions/day	193 (93.2)	14 (6.8)	
**Vegetables (daily portions)**			0.177
<4 portions/day	352 (95.7)	16 (4.3)	
4–7 portions/day	416 (93.5)	29 (6.5)	
≥8 portions/day	222 (92.1)	19 (7.9)	
**Fruits (daily portions)**			0.850
<3 portions/day	281 (94.3)	17 (5.7)	
3–5 portions/day	417 (94.1)	26 (5.9)	
≥4 portions/day	292 (93.3)	21 (6.7)	
**Nuts and seeds (weekly portions)**			0.838
<4 portions/week	755 (93.7)	51 (6.3)	
4 portions/week—1/day	174 (95.1)	9 (4.9)	
≥1.5 portions/day	61 (93.8)	4 (6.2)	
**Vegetable oils, avocado, olives (daily portions)**			0.922
≤2 portions/day	578 (93.7)	39 (6.3)	
>2–4 portions/day	331 (94.3)	20 (5.7)	
>4 portions/day	81 (94.2)	5 (5.8)	
**Dairy products (daily portions)**			0.743
None	192 (95.0)	10 (5.0)	
≤2 portions/day	566 (93.6)	39 (6.4)	
>2 portions/day	232 (93.9)	15 (6.1)	
**Eggs (daily portions)**			>0.999
None	77 (93.9)	5 (6.1)	
≤1 portion/day	520 (93.9)	34 (6.1)	
>1 portion/day	393 (94.0)	25 (6.0)	
**Sweets (weekly portions)**			0.141
<2 portions/week	482 (95.4)	23 (4.6)	
2–5 portions/week	373 (92.6)	30 (7.4)	
>5 portions/week	135 (92.5)	11 (7.5)	
**Reliable sources of vitamin B12 (daily portions)**			0.005
<1 portion/day	215 (96.0)	9 (4.0)	
1 portion/day	485 (95.3)	24 (4.7)	
≥2 portions/day	290 (90.3)	31 (9.7)	
**Meat consumption (weekly frequency)**			0.265
None	56 (93.3)	4 (6.7)	
<1 time/month—1/week	469 (95.1)	24 (4.9)	
≥2 times/week	465 (92.8)	36 (7.2)	
**Physical activity (daily minutes)**			0.574
None	235 (94.0)	15 (6.0)	
<30 min moderate/< 15 min vigorous	404 (93.1)	30 (6.9)	
≥30 min moderate/≥ 15 min vigorous	351 (94.9)	19 (5.1)	
**Water intake (250 mL glasses/day)**			0.006
<4	205 (96.2)	8 (3.8)	
4–7	491 (95.2)	25 (4.8)	
≥8	294 (90.5)	31 (9.5)	
**Sunlight exposure (daily minutes, 11 a.m.–1 p.m.)**			0.148
<5 min/day	319 (91.9)	28 (8.1)	
5–9 min/day	400 (95.2)	20 (4.8)	
≥10 min/day	271 (94.4)	16 (5.6)	
**Lifestyle score (M ± SD)**	26.8 (4.0)	27.7 (3.4)	0.050
**Categorical lifestyle score**			0.179
<27	488 (94.9)	26 (5.1)	
≥27	502 (93.0)	38 (7.0)	

**Note.** n (%); Mean (SD); ^2^ chi-square test of independence; Wilcoxon rank-sum test.

**Table 5:** Lifestyle Components According to Waist Circumference Categories.

Characteristic	Normal (N = 669)	Elevated (N = 385)	*p*-Value ^2^
**Whole grains (daily portions)**	n (%)	n (%)	0.009
<3 portions/day	336 (59.8)	226 (40.2)	
3–5 portions/day	277 (66.3)	141 (33.7)	
≥6 portions/day	56 (75.7)	18 (24.3)	
**Legumes (daily portions)**			0.887
<1 portion/day	208 (64.4)	115 (35.6)	
1–2 portions/day	332 (63.4)	192 (36.6)	
≥3 portions/day	129 (62.3)	78 (37.7)	
**Vegetables (daily portions)**			0.465
<4 portions/day	228 (62.0)	140 (38.0)	
4–7 portions/day	292 (65.6)	153 (34.4)	
≥8 portions/day	149 (61.8)	92 (38.2)	
**Fruits (daily portions)**			0.290
<3 portions/day	179 (60.1)	119 (39.9)	
3–5 portions/day	283 (63.9)	160 (36.1)	
≥4 portions/day	207 (66.1)	106 (33.9)	
**Nuts and seeds (weekly portions)**			0.370
<4 portions/week	520 (64.5)	286 (35.5)	
4 portions/week—1/day	112 (61.2)	71 (38.8)	
≥1.5 portions/day	37 (56.9)	28 (43.1)	
**Vegetable oils, avocado, olives (daily portions)**			0.680
≤2 portions/day	392 (63.5)	225 (36.5)	
>2–4 portions/day	226 (64.4)	125 (35.6)	
>4 portions/day	51 (59.3)	35 (40.7)	
**Dairy products (daily portions)**			0.363
None	123 (60.9)	79 (39.1)	
≤2 portions/day	395 (65.3)	210 (34.7)	
>2 portions/day	151 (61.1)	96 (38.9)	
**Eggs (daily portions)**			0.152
None	56 (68.3)	26 (31.7)	
≤1 portion/day	362 (65.3)	192 (34.7)	
>1 portion/day	251 (60.0)	167 (40.0)	
**Sweets (weekly portions)**			0.361
<2 portions/week	325 (64.4)	180 (35.6)	
2–5 portions/week	246 (61.0)	157 (39.0)	
>5 portions/week	98 (67.1)	48 (32.9)	
**Reliable sources of vitamin B12 (daily portions)**			0.852
<1 portion/day	142 (63.4)	82 (36.6)	
1 portion/day	327 (64.2)	182 (35.8)	
≥2 portions/day	200 (62.3)	121 (37.7)	
**Meat consumption (weekly frequency)**			0.332
None	41 (68.3)	19 (31.7)	
<1 time/month—1/week	302 (61.3)	191 (38.7)	
≥2 times/week	326 (65.1)	175 (34.9)	
**Physical activity (daily minutes)**			<0.001
None	136 (54.4)	114 (45.6)	
<30 min moderate/< 15 min vigorous	271 (62.4)	163 (37.6)	
≥30 min moderate/≥ 15 min vigorous	262 (70.8)	108 (29.2)	
**Water intake (250 mL glasses/day)**			0.182
<4	143 (67.1)	70 (32.9)	
4–7	332 (64.3)	184 (35.7)	
≥8	194 (59.7)	131 (40.3)	
**Sunlight exposure (daily minutes, 11 a.m.–1 p.m.)**			0.377
<5 min/day	210 (60.5)	137 (39.5)	
5–9 min/day	273 (65.0)	147 (35.0)	
≥10 min/day	186 (64.8)	101 (35.2)	
**Lifestyle score (M ± SD)**	26.9 (3.9)	26.7 (4.0)	0.246
**Categorical lifestyle score**			0.291
<27	318 (61.9)	196 (38.1)	
≥27	351 (65.0)	189 (35.0)	

**Note.** n (%); Mean (SD); ^2^ chi-square test of independence; Wilcoxon rank-sum test.

**Table 6:** Association Between Lifestyle Components and Elevated Waist Circumference: Crude and Adjusted Poisson Regression Models.

Characteristic	Crude Regression			Adjusted Regression *		
	PR	95% CI	*p*-Value	PR	95% CI	*p*-Value
**Whole grains (daily portions)**						
<3 portions/day	1	–	–	1	–	–
3–5 portions/day	1.07	0.65–1.74	0.793	1.08	0.66–1.78	0.755
≥6 portions/day	0.67	0.21–2.13	0.497	0.66	0.21–2.09	0.479
**Legumes (daily portions)**						
<1 portion/day	1	–	–	1	–	–
1–2 portions/day	1.95	1.04–3.68	0.039	1.98	1.06–3.72	0.032
≥3 portions/day	1.82	0.86–3.86	0.118	1.81	0.85–3.83	0.121
**Vegetables (daily portions)**						
<4 portions/day	1	–	–	1	–	–
4–7 portions/day	1.50	0.83–2.72	0.182	1.50	0.82–2.73	0.184
≥8 portions/day	1.81	0.95–3.46	0.070	1.79	0.95–3.39	0.074
**Fruits (daily portions)**						
<3 portions/day	1	–	–	1	–	–
3–5 portions/day	1.03	0.57–1.86	0.925	1.02	0.57–1.85	0.938
≥4 portions/day	1.18	0.63–2.19	0.608	1.18	0.63–2.19	0.602
**Nuts and seeds (weekly portions)**						
<4 portions/week	1	–	–	1	–	–
4 portions/week—1/day	0.78	0.39–1.55	0.474	0.76	0.37–1.53	0.438
≥1.5 portions/day	0.97	0.36–2.61	0.956	0.96	0.36–2.55	0.937
**Vegetable oils, avocado, olives (daily portions)**						
≤2 portions/day	1	–	–	1	–	–
>2–4 portions/day	0.90	0.53–1.52	0.697	0.90	0.53–1.52	0.692
>4 portions/day	0.92	0.37–2.27	0.856	0.90	0.37–2.19	0.817
**Dairy products (daily portions)**						
None	1	–	–	1	–	–
≤2 portions/day	1.30	0.66–2.56	0.444	1.32	0.68–2.59	0.413
>2 portions/day	1.23	0.56–2.67	0.607	1.23	0.56–2.66	0.608
**Eggs (daily portions)**						
None	1	–	–	1	–	–
≤1 portion/day	1.01	0.41–2.50	0.989	1.03	0.42–2.56	0.942
>1 portion/day	0.98	0.39–2.49	0.968	0.97	0.39–2.46	0.957
**Sweets (weekly portions)**						
<2 portions/week	1	–	–	1	–	–
2–5 portions/week	1.63	0.96–2.77	0.068	1.67	0.99–2.83	0.054
>5 portions/week	1.65	0.83–3.31	0.155	1.66	0.82–3.35	0.159
**Reliable sources of vitamin B12 (daily portions)**						
<1 portion/day	1	–	–	1	–	–
1 portion/day	1.17	0.55–2.48	0.676	1.19	0.56–2.51	0.654
≥2 portions/day	2.40	1.17–4.95	0.017	2.39	1.16–4.91	0.018
**Meat consumption (weekly frequency)**						
None	1	–	–	1	–	–
<1 time/month—1/week	0.73	0.26–2.03	0.547	0.74	0.27–2.07	0.570
≥2 times/week	1.08	0.40–2.92	0.883	1.09	0.40–2.97	0.862
**Physical activity (daily minutes)**						
None	1	–	–	1	–	–
<30 min moderate/<15 min vigorous	1.15	0.63–2.10	0.644	1.18	0.64–2.19	0.600
≥30 min moderate/≥15 min vigorous	0.86	0.44–1.65	0.643	0.89	0.44–1.82	0.756
**Water intake (250 mL glasses/day)**						
<4	1	–	–	1	–	–
4–7	1.29	0.59–2.81	0.522	1.32	0.60–2.91	0.488
≥8	2.54	1.19–5.42	0.016	2.61	1.20–5.66	0.015
**Sunlight exposure (daily minutes, 11 a.m.–1 p.m.)**						
<5 min/day	1	–	–	1	–	–
5–9 min/day	0.59	0.34–1.03	0.063	0.59	0.34–1.02	0.059
≥10 min/day	0.69	0.38–1.25	0.222	0.69	0.38–1.26	0.229
**Lifestyle score (categorical)**						
<27	1	–	–	1	–	–
≥27	1.39	0.86–2.26	0.181	1.40	0.86–2.29	0.175

**Note.** PR = prevalence ratio; 95% CI = 95% confidence interval. Crude and adjusted PRs were estimated using Poisson regression with robust standard errors; statistical significance was set at *p* < 0.05. * Adjusted model: multivariable model adjusted for prespecified covariates (e.g., age, sex, and other variables included in the model; see Methods).

**Table 7:** Robust Poisson Regression Models for the Association Between Lifestyle Components and Elevated Waist Circumference.

Characteristic	Crude Regression			Adjusted Regression *		
	PR	95% CI	*p*-Value	PR	95% CI	*p*-Value
**Whole grains (daily portions)**						
<3 portions/day	1.00			1.00		
3–5 portions/day	0.84	0.71–0.99	0.040	0.86	0.73–1.02	0.091
≥6 portions/day	0.60	0.40–0.92	0.017	0.60	0.40–0.91	0.015
**Legumes (daily portions)**						
<1 portion/day	1.00			1.00		
1–2 portions/day	1.03	0.86–1.24	0.761	1.06	0.88–1.27	0.560
≥3 portions/day	1.06	0.84–1.33	0.627	1.06	0.84–1.33	0.622
**Vegetables (daily portions)**						
<4 portions/day	1.00			1.00		
4–7 portions/day	0.90	0.75–1.09	0.278	0.92	0.76–1.10	0.355
≥8 portions/day	1.00	0.82–1.23	0.974	0.99	0.80–1.21	0.893
**Fruits (daily portions)**						
<3 portions/day	1.00			1.00		
3–5 portions/day	0.90	0.75–1.09	0.291	0.91	0.76–1.09	0.312
≥4 portions/day	0.85	0.69–1.04	0.121	0.85	0.69–1.04	0.119
**Nuts and seeds (weekly portions)**						
<4 portions/week	1.00			1.00		
4 portions/week—1/day	1.09	0.89–1.34	0.392	1.09	0.89–1.34	0.403
≥1.5 portions/day	1.21	0.90–1.63	0.197	1.23	0.91–1.64	0.175
**Vegetable oils, avocado, olives (daily portions)**						
≤2 portions/day	1.00			1.00		
>2–4 portions/day	0.98	0.82–1.16	0.791	0.98	0.82–1.17	0.827
>4 portions/day	1.12	0.85–1.47	0.435	1.09	0.83–1.43	0.549
**Dairy products (daily portions)**						
None	1.00			1.00		
≤2 portions/day	0.89	0.72–1.09	0.251	0.91	0.74–1.11	0.347
>2 portions/day	0.99	0.79–1.25	0.958	0.99	0.79–1.25	0.965
**Eggs (daily portions)**						
None	1.00			1.00		
≤1 portion/day	1.09	0.78–1.53	0.606	1.12	0.80–1.57	0.511
>1 portion/day	1.26	0.90–1.77	0.181	1.25	0.89–1.75	0.201
**Sweets (weekly portions)**						
<2 portions/week	1.00			1.00		
2–5 portions/week	1.09	0.92–1.29	0.303	1.11	0.94–1.31	0.229
>5 portions/week	0.92	0.71–1.20	0.542	0.91	0.70–1.18	0.480
**Reliable sources of vitamin B12 (daily portions)**						
<1 portion/day	1.00			1.00		
1 portion/day	0.98	0.79–1.20	0.825	1.00	0.81–1.22	0.966
≥2 portions/day	1.03	0.82–1.29	0.796	1.03	0.83–1.29	0.782
**Meat consumption (weekly frequency)**						
None	1.00			1.00		
<1 time/month—1/week	1.22	0.83–1.80	0.308	1.28	0.87–1.87	0.213
≥2 times/week	1.10	0.75–1.63	0.622	1.15	0.78–1.69	0.485
**Physical activity (daily minutes)**						
None	1.00			1.00		
<30 min moderate/<15 min vigorous	0.82	0.69–0.99	0.036	0.85	0.71–1.02	0.087
≥30 min moderate/≥15 min vigorous	0.64	0.52–0.79	<0.001	0.69	0.56–0.86	0.001
**Water intake (250 mL glasses/day)**						
<4	1.00			1.00		
4–7	1.09	0.87–1.36	0.476	1.12	0.89–1.40	0.333
≥8	1.23	0.97–1.55	0.086	1.28	1.01–1.62	0.039
**Sunlight exposure (daily minutes, 11 a.m.–1 p.m.)**						
<5 min/day	1.00			1.00		
5–9 min/day	0.89	0.74–1.07	0.200	0.90	0.75–1.09	0.286
≥10 min/day	0.89	0.73–1.09	0.269	0.93	0.76–1.14	0.497
**Lifestyle score (categorical)**						
<27	1.00			1.00		
≥27	0.92	0.78–1.08	0.291	0.94	0.80–1.10	0.448

**Note.** PR = prevalence ratio; 95% CI = 95% confidence interval. Crude and adjusted PRs were estimated using Poisson regression with robust standard errors; statistical significance was set at *p* < 0.05. * Adjusted model: multivariable model adjusted for prespecified covariates (e.g., age, sex, and other variables included in the model; see Methods).

## Data Availability

The datasets generated and analyzed during the current study are not publicly available due to ethical and legal restrictions imposed by the Research Ethics Committee of Universidad Peruana Unión. Data may, however, be made available from the corresponding author upon reasonable request and with permission from the Research Ethics Committee.
